# Morphometric Evaluation of Maggot Debridement Therapy on Healing Outcomes in Chronic Wounds

**DOI:** 10.3390/jcm15124490

**Published:** 2026-06-10

**Authors:** Emrah Altuntas, Bahadir Yazicioglu, Orhan Bas, Onur Ozturk

**Affiliations:** 1 Department of Anatomy, Faculty of Medicine, Samsun University, Samsun 55080, Turkey; orhanbas55@hotmail.com; 2 Department of Family Medicine, Samsun Education and Research Hospital, Samsun 55090, Turkey; bahadiryazicioglu@gmail.com; 3 Department of Family Medicine, Faculty of Medicine, Amasya University, Amasya 05100, Turkey; onur.ozturk@amasya.edu.tr

**Keywords:** Maggot debridement therapy, wound healing, chronic wounds, wound area reduction, morphometric analysis

## Abstract

**Background/Objectives**: Maggot debridement therapy (MDT) is an established biodebridement modality in chronic wound management; however, quantitative evidence regarding its effects on wound-healing dynamics remains limited. This study aimed to evaluate morphometric healing changes in chronic wounds treated with MDT using quantitative image-based analysis. **Methods**: This retrospective observational study analyzed archival records of patients who underwent MDT between 1 January 2024, and 1 January 2025. Wound images acquired according to a standardized clinical photography protocol were analyzed in ImageJ software (version 1.53k) after scale calibration. Lesion areas were measured in a blinded manner by two independent anatomy specialists. Morphometric data were analyzed using R, and healing trajectories were evaluated using the Kaplan–Meier method. **Results**: A total of 95 chronic wound cases were included. The mean age was 65 ± 11 years, and 73% of patients were male. Most lesions were localized to the foot (91%), and 40% were classified as stage 3 wounds. A total of 294 MDT sessions were performed. The mean wound-area reduction per session was 9.7% (median 6.6%). Wound-area reduction differed significantly across treatment sessions (H = 14; *p* = 0.008), with the greatest improvement during the first three sessions; pairwise analysis showed a significant difference between sessions 1 and 3 (*p* = 0.029). Approximately 18% of cases achieved ≥50% wound-area reduction, with a median of eight sessions required to reach this threshold. Age and sex were not significantly associated with healing outcomes. **Conclusions**: MDT facilitates measurable reductions in wound area and contributes to the healing process in chronic wounds. The findings suggest that the therapeutic effect of MDT may be more pronounced during the early treatment sessions and may help optimize treatment planning in chronic wound management.

## 1. Introduction

Wound healing is a progressive and dynamic tissue repair process wherein anatomical and functional continuity are restored following disruption of the structural integrity of the epidermal, dermal, and subdermal tissues. This restoration involves coordinated participation of cellular and stromal components, extracellular matrix remodeling, and local microvascular perfusion [1].

Chronic wounds, in which this process is impaired, are recognized as significant clinical and anatomical challenges because of their multifactorial pathophysiology and prolonged healing times [2,3]. In chronic wounds, beyond superficial tissue damage, the dermal–subdermal tissue architecture, extracellular matrix organization, and the function of the local vascular network are markedly affected [4,5,6].

In addition to conventional surgical and pharmacological approaches for managing chronic wounds, maggot debridement therapy (MDT), a biologically based intervention within integrative medicine, has emerged as a cost-effective therapeutic approach [7]. MDT is based on the application of sterile larvae of *Lucilia (Phaenicia) sericata* to the wound bed to achieve biological debridement of necrotic tissue [8,9]. The clinical and economic burden of chronic wounds has become more prominent with population aging and the increasing prevalence of diabetes mellitus, peripheral vascular disease, and other chronic comorbidities. Because these wounds often require prolonged care and are accompanied by recurrent infection, pain, reduced mobility, impaired quality of life, and substantial healthcare costs, accessible and cost-conscious therapeutic strategies remain important in chronic wound management [10,11].

The mechanism of action of MDT involves the removal of necrotic tissue and reduction of microbial burden through larval secretion of proteolytic enzymes (including leucine aminopeptidase, collagenolytic enzymes, and other related proteases), antimicrobial compounds, and ammonia-containing substances, thereby establishing a biochemical microenvironment that promotes wound healing [12,13]. This mechanism of action has been shown to accelerate the wound-healing process and support the restoration of tissue structural integrity and functionality in a range of chronic and complicated wounds, including diabetic foot ulcers and venous stasis ulcers, non-diabetic neuropathic foot ulcers, pressure ulcers, wounds refractory to healing after trauma, arterial-ischemic ulcers, postoperative wound areas, osteomyelitis, and necrotizing fasciitis [14]. Recent evidence suggests that the role of MDT is not limited to biodebridement alone. In chronic wounds, bacterial biofilms may persist within the wound bed and contribute to sustained inflammation, reduced antimicrobial susceptibility, and delayed tissue repair [15]. In this context, MDT and larval-derived products have been reported to decrease microbial burden, affect biofilm organization, and create local wound-bed conditions that may support granulation tissue formation and subsequent repair [11,16]. However, despite these potential benefits, MDT is still not routinely used in many clinical settings. This limited use may be related to patient acceptance, pain or discomfort during treatment, access to sterile larvae, issues related to cost, training, and accessibility, and insufficient familiarity with the method among healthcare professionals [10,11].

Quantitative monitoring of changes in wound surface area during chronic wound management is considered one of the key anatomical parameters that reveal the morphological course of tissue repair [17]. In this context, wound photography provides a non-invasive, reproducible documentation method that allows delineation of lesion margins and comparative assessment of area changes over time [18]. Because MDT-related wound-bed changes may occur progressively across treatment sessions, standardized photographic assessment provides a practical method for evaluating the temporal pattern of wound-area reduction.

Several previous studies have demonstrated the clinical efficacy of MDT with respect to wound debridement, infection control, and promotion of tissue repair [7,12,19]. However, most published studies have focused on clinical outcomes, patient-reported measures, or overall healing rates, whereas relatively few investigations have quantitatively evaluated the session-based anatomical and morphometric dynamics of wound-bed remodeling during MDT using standardized image-based measurements. Therefore, the present study aimed to characterize inter-session morphological changes in chronic wounds treated with MDT using standardized quantitative photographic analysis. In addition, on the basis of anatomical and morphometric parameters, we determined the temporal distribution of the therapeutic effect and the session interval in which MDT achieves maximal efficacy during reorganization of wound tissue architecture.

## 2. Materials and Methods

### 2.1. Study Design and Ethical Approval

This retrospective observational study was conducted using archival records of patients who underwent MDT at a tertiary care integrative medicine center between 1 January 2024, and 1 January 2025. The study was conducted in accordance with the Declaration of Helsinki and approved by the Kayseri City Hospital Clinical Research Ethics Committee (Approval No. 86; approved on 14 February 2025).

### 2.2. Study Population and Data Collection

Patients with complete sets of lesion images were eligible for inclusion. Cases were included if the image set contained a pre-treatment baseline image, at least one follow-up image after MDT, a visible millimeter-grid reference for scale calibration, and wound margins that could be reliably delineated. Cases with incomplete image sets, inadequate image quality, unavailable calibration reference, or missing essential archive data were excluded. A total of 95 lesions (one per patient) were included in the analysis. For a patient who presented with more than one lesion, only the largest lesion was used for analysis.

Wound images had previously been acquired by academic personnel trained in the standard photography protocol within the Departments of Family Medicine and Anatomy as part of routine clinical care and stored in a digital archive. All images were captured perpendicular to the wound surface without the application of digital filters or post-processing, under uniform lighting conditions, using a full-frame DSLR camera (Nikon D810; Nikon Corporation, Tokyo, Japan) equipped with a 24–100 mm lens. To ensure measurement accuracy, the millimeter-grid paper present in the images was used as a reference for scale calibration during the analysis phase.

Demographic data, comorbid diseases, lesion localization, wound stage, and the number of treatment sessions were obtained retrospectively from hospital archive records. The sample size was determined by including all eligible cases that underwent MDT during the study period and met the inclusion criteria.

### 2.3. MDT Protocol

Larvae production in Turkey is carried out only at the Microbiology laboratory of Istanbul University-Cerrahpaşa (Istanbul, Turkey); a protocol has been established with our institution, and the larvae are delivered to us via cargo. The transfer is completed in 8–10 h. Within the clinical treatment program, all cases were treated with sterile *L. sericata* larvae at developmental stages 1–2. The larvae were applied to the wound surface using the free-range (direct) application technique at a density of 8–10 larvae/cm^2^, consistent with the biodebridement density range recommended in the literature [9]. Before application, the area to be treated was cleansed with distilled water. Without applying any topical agent, the larvae were placed under sterile conditions and covered with dry gauze. After larval removal, the wound area was again cleansed with distilled water and covered with semi-moist gauze. While the larvae remained in place, the limb could be moved provided that no external pressure was applied to the treated area. Larvae were removed from the wound bed approximately 72 h after application. For the present analysis, one MDT session was defined as one larval application period lasting approximately 72 h. The image obtained before the first larval application was defined as session 0, and subsequent images obtained during documented follow-up visits after MDT applications were assigned consecutive session numbers. Between sessions, the wound bed was evaluated clinically for the presence of necrotic tissue, signs of infection, and development of granulation tissue; necessary dressing changes were performed. During the intervals between MDT sessions, standard dressing care included the application of creams containing *Hamamelis virginiana* and zinc for tissue-protective and regenerative support, together with alginate hydrogel to facilitate removal of devitalized tissue. MDT applications were repeated until there was a marked reduction of necrotic tissue, development of healthy granulation tissue, and evidence of epithelialization.

### 2.4. Image Analysis, Lesion Measurement, and Interobserver Agreement

**Figure 1:**
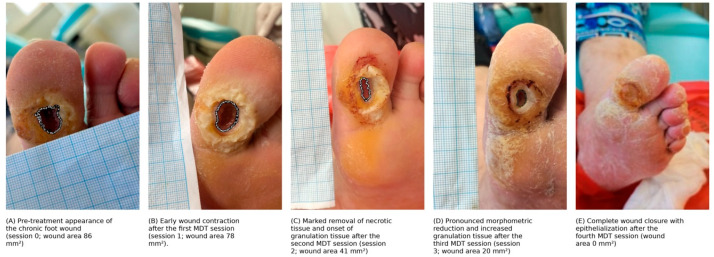
Serial morphometric changes across consecutive sessions in a representative chronic foot wound treated with maggot debridement therapy (MDT). Scale calibration was performed using the millimetric reference.

Wound images were exported to ImageJ software (version 1.53k; NIH, Bethesda, MD, USA) and analyzed retrospectively. Images were calibrated using the Set Scale tool, with millimeter-grid paper present at the lesion margins serving as the scale reference. Lesion boundaries were delineated manually using a planimetric approach with the Polygon Selection tool, and wound surface area was calculated in square millimeters (mm^2^) (Figure 1). Percentage wound-area reduction was calculated using the following formula: [(reference wound area − follow-up wound area)/reference wound area] × 100. For session-based comparisons, the preceding session was used as the reference, whereas baseline wound area was used as the reference for Kaplan–Meier analyses evaluating time to ≥30% and ≥50% wound-area reduction.

All morphometric measurements were performed in a blinded manner by two experienced anatomy specialists, independently of patient identifiers and clinical data. Interobserver agreement was assessed using a two-way mixed-effects model in R software (version 4.5.2; R Foundation for Statistical Computing, Vienna, Austria). The absolute agreement type intraclass correlation coefficient (ICC) was calculated using the ICC function from the psych package and interpreted according to the criteria proposed by Koo and Li [20].

### 2.5. Wound Staging

Pressure (decubitus) ulcers were staged according to the five-tier classification system defined by the American and European Pressure Ulcer Advisory Panels [21]. Staging was performed independently by three investigators experienced in chronic wound assessment. The final stage classification was assigned by consensus of at least two evaluators. Wound stage was recorded separately for each patient in the pre-treatment and post-treatment periods.

### 2.6. Statistical Analysis

Statistical analyses were conducted using R software (version 4.5.2; R Foundation for Statistical Computing, Vienna, Austria). Continuous variables were summarized as mean ± standard deviation and median (minimum–maximum). Times to ≥30% and ≥50% reductions in wound area relative to baseline were evaluated using the Kaplan–Meier method. The treatment session at which the threshold was reached was defined as the “event”; patients who did not reach the threshold were censored at the last follow-up. Between-session differences in wound-area reduction were assessed using the Kruskal–Wallis test, followed by pairwise comparisons with the Mann–Whitney U test with Bonferroni correction. Logistic regression analysis was performed to evaluate the effects of age and sex on healing outcomes. For logistic regression analyses, achievement of ≥30% and ≥50% wound-area reduction was coded separately as binary outcomes, with age and sex entered as independent variables. Odds ratios and 95% confidence intervals were reported. All statistical tests were two-tailed. Statistical significance was set at *p* < 0.05.

## 3. Results

A total of 95 cases were included in the study. Of them, 73% (*n* = 69) of patients were male. The mean age was 65 ± 11 years. A total of 294 MDT treatment sessions were performed. Most lesions were localized to the foot (91%; *n* = 86).

At initial presentation to the clinic (session 0), 40% (*n* = 38), 21% (*n* = 20), and 19% (*n* = 18) of lesions were classified as stages 3, 2, and 4, respectively. Twenty percent (*n* = 19) of lesions were categorized as an unspecified stage, and no stage 1 wounds were identified.

During treatment sessions, wound surface areas exhibited a statistically significant decreasing trend (H = 14; *p* = 0.008). Mean and median reductions per session were 9.7% and 6.6%, respectively. Wound-area reduction after the first session was modest, whereas morphometric improvement became more pronounced from the second and third sessions onward. Although variability increased in later sessions in parallel with declining patient numbers, wound-area reduction persisted (Table 1).

When stage regression by at least one category was assessed across sessions, stage-based improvement was most pronounced during early sessions. The highest rate of stage regression was observed after the first session, followed by the third and fourth sessions. Limited stage regression was observed in later sessions (Table 1).

Pairwise comparisons (Table 2) indicated that wound reduction between sessions 1 and 3 was statistically significant (*p* = 0.029). No significant differences were observed among other session pairs.

When times to ≥1-stage wound improvement were evaluated using the Kaplan–Meier method, the median time to improvement was two sessions. Most cases that achieved stage improvement reached this threshold within the first 2–3 treatment sessions. Subsequent sessions yielded markedly fewer new stage-based improvements. These findings indicate that MDT exerts a particularly pronounced clinical effect on wound stage during the early phase of treatment.

When the relationship between stage regression and percentage wound-area reduction was assessed, the median area reduction for the 13 wounds that regressed from stage 4 to 3 was 8.5%. The median area reduction for the 27 wounds that regressed from stage 3 to 2 was 18%. The median area reduction for the seven wounds that regressed from stage 2 to 1 was 34%. However, no significant difference in the percentage area reduction was observed across these stage-regression groups (H = 3; *p* = 0.22).

Stage-regression comparisons between paired groups did not reveal statistically significant differences (Table 3).

**Figure 2:**
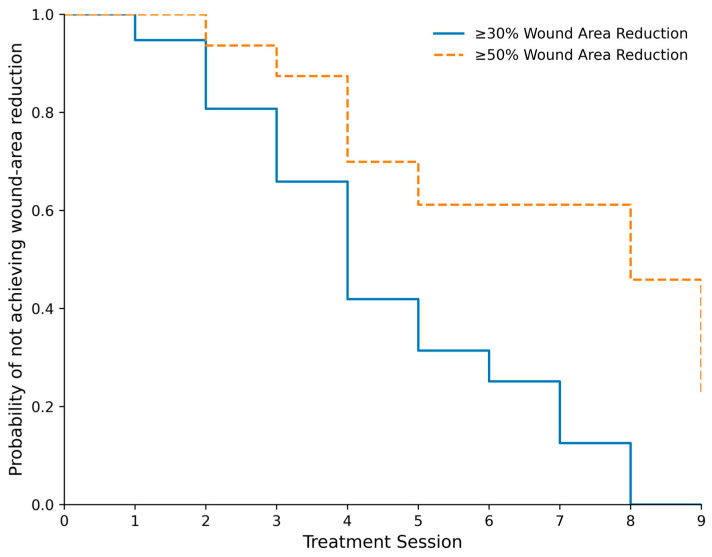
Kaplan–Meier curves demonstrating the proportions of lesions achieving ≥30% and ≥50% wound-area reductions across treatment sessions. The corresponding number-at-risk data are presented in Table 4.

When the time to ≥30% wound-area reduction relative to baseline was evaluated, the median time was four sessions (Figure 2). Moreover, 36% (*n* = 34) of cases reached this threshold. A pronounced decline was observed in the proportion of lesions achieving the 30% reduction threshold over the first four sessions. For time to ≥50% wound-area reduction, the median time was eight sessions, and only 18% (*n* = 17) of cases achieved this level (Figure 2). Kaplan–Meier analysis indicated that the probability of achieving a 50% wound-area reduction was relatively low, and it typically occurred during later treatment sessions. The corresponding number-at-risk data, including events, censored observations, and Kaplan–Meier-estimated cumulative probabilities, are presented in Table 4.

There was no statistically significant difference in wound localization by sex (*p* > 0.05). In logistic regression analyses, neither sex nor age had a statistically significant effect on the likelihood of achieving either a 30% or a 50% wound-area reduction (*p* > 0.05) (Table 5).

## 4. Discussion

This study investigated MDT—a treatment whose efficacy in difficult-to-manage clinical wound scenarios has been documented—from an anatomical perspective. The results provide evidence of MDT’s beneficial effect using quantitative morphometric analyses.

It is estimated that approximately 2% of the population in developing countries suffers from chronic wounds, and this prevalence increases with population aging [8]. Despite advances in wound-care products that can modulate and accelerate wound healing—a highly complex physiological process particularly vulnerable in the presence of comorbid conditions—chronic wounds remain a significant public health problem. Therefore, clinicians implement various therapeutic approaches guided by evidence-based medicine. MDT is an FDA-cleared therapeutic method [22] that is referenced in the 2023 guideline developed by the World Health Summit for the management of pressure ulcers, particularly owing to its debridement and antibacterial properties [23]. In Turkey, MDT is a reasonably priced healthcare practice whose pricing is regulated by the Ministry of Health; however, it is not currently included within the reimbursement scope of the Social Security Institution. As a result, despite MDT’s well-documented efficacy in the literature, patient access to the therapy is limited. In addition, the insufficient number of provider centers and trained professionals further restricts access to this therapeutic option [24]. Given that our data derive from this narrowly accessible service spectrum, the present study seeks to fill an important gap in the literature.

Change in wound surface area over time is a useful parameter for evaluating the effectiveness of therapies in chronic wound management [17]. Wound photography is widely used for delineating lesion morphology, comparing treatment-related changes over time, and providing clinical documentation [18]. The present study employed professional photographic equipment and objective surface-area measurement, thereby minimizing subjective bias.

A search of PubMed using the filters “Humans,” “Clinical Trial,” “Meta-Analysis,” “Randomized Controlled Trial,” and “Systematic Review” yielded 12 MDT studies published in the past ten years. A review of these reports provides data on wound healing following MDT and on comparisons between MDT and alternative therapies; however, there is no clear or sufficient information in the literature regarding the number of sessions required or the amount of improvement observed at each session. An earlier study of 54 wounds treated with *L. sericata* larvae reported that, on average, 2.4 sessions were required to achieve a significant reduction in necrotic tissue [25]. According to the morphometric analyses in the present study, wound improvement was more pronounced in early treatment sessions, with the rate of improvement decelerating in later sessions while remaining cumulative. A plausible explanation for the greater improvement observed during the first three MDT sessions is the rapid removal of necrotic tissue and reduction of microbial burden during the early phase of treatment. Previous studies have shown that larval excretions/secretions contain proteolytic enzymes capable of selectively degrading devitalized tissue while preserving viable structures, thereby facilitating wound-bed preparation and granulation tissue formation [26,27]. In addition, MDT has been reported to reduce microbial burden and disrupt bacterial biofilms, while its secretions have been suggested to modulate local inflammatory and tissue-repair responses, including processes related to granulation and extracellular matrix remodeling [28]. These complementary effects may contribute to the accelerated early healing response observed in the present study. As the necrotic tissue burden decreases and the wound progresses toward later stages of repair, the rate of measurable surface-area reduction may become less pronounced despite ongoing tissue remodeling and healing activity. This pattern might be attributed to the multifactorial nature of wound closure. Heterogeneity in glycemic control among our predominantly diabetic cohort and the chronicity of underlying disease render a healing trajectory different from that of uncomplicated acute wounds. Patients who achieved approximately one-third reductions in wound area reached that threshold within approximately one month. Patients who achieved roughly 50% area reduction reached that threshold within approximately two months. These observations contribute novel, evidence-based information to the existing literature. Under physiological conditions, uncomplicated wound healing is typically completed within 3 weeks [29]. In the presence of comorbidities such as diabetes, wound healing may require months or even years, and, in some cases, wounds fail to heal and progress to amputation [30]. Conversely, MDT has been reported as a cost-effective intervention for wound debridement and infection control and may prevent amputations in up to approximately 85% of cases [31]. To optimize outcomes, MDT was used in combination with standardized surgical debridement and conventional pharmacological strategies where clinically indicated. Adjunctive interventions included debridement gels, topical and systemic antimicrobial agents, and formulations intended to promote granulation and re-epithelialization. Comorbid conditions (for example, diabetes, arterial and venous disease, active infection, and tobacco use) were managed medically according to patient needs. These adjunctive therapies are known to contribute to wound healing, and larger-scale, detailed investigations are required to delineate their relative contributions. A practical advantage of MDT is its applicability across diverse patient populations with open wounds without introducing substantial polypharmacy risk [2].

In our statistical analyses, neither patient sex nor age was a significant determinant of wound-area reduction following MDT. In a previous study conducted in Turkey, Hakyemez et al. (2024) reported that, among patients with pressure ulcers in Turkey, the majority were female and the mean age of the cohort was 71 years [32]. The mean age of our cohort was comparable; however, the sex distribution differed. The same study also reported that 40% of their cohort had stage-3 pressure ulcers, and approximately half of the ulcers were sacral. In contrast, our cohort exhibited a similar stage distribution but a substantially higher frequency of foot-localized wounds. Several factors may account for these demographic and clinical differences. The referenced study was conducted among inpatients, whereas the patients in our series were registered at a fee-based, outpatient public clinic. A separate outpatient wound-clinic study reported a case mix similar to ours—predominantly male patients in their 60 s with diabetic foot ulcers [33]. This finding may explain the diversity of patients’ presenting complaints. Chronic foot wounds are commonly secondary to diabetes [34], with wounds typically originating in the feet; over time, as additional complications develop, patients may become bedridden and subsequently develop sacral pressure ulcers. Additionally, higher average life expectancy in women and the older age of hospitalized patients may contribute to a greater prevalence of sacral ulcers in women and foot ulcers in men [35]. Although male patients constituted the majority of the study population, sex was not significantly associated with the likelihood of achieving either 30% or 50% wound-area reduction. However, because the vast majority of lesions were localized to the foot and only a small number of wounds originated from other anatomical regions, the present cohort was not optimally suited to evaluate potential interactions between sex, wound location, and healing outcomes. Future studies including larger and more anatomically diverse patient populations are needed to further clarify these relationships.

Numerous factors influence tissue tolerance and wound-healing capacity, including age, nutritional status, concomitant medications, comorbid conditions, and tobacco exposure [2]. Nearly all patients in our cohort had diabetes. Diabetic foot ulcers, a microvascular complication of diabetes and one of the most common causes of morbidity and mortality worldwide, may affect up to 15% of people with diabetes at least once during their lifetime [36]. This epidemiologic burden underscores the clinical importance of studies that evaluate effective therapies for diabetic foot wounds. Implementation of MDT in our predominantly diabetic cohort produced favorable outcomes according to morphometric criteria.

### Limitations

This study has several limitations. MDT was applied to all open wounds in the clinical setting where treatments were performed; therefore, a concurrent control group is lacking. Consequently, MDT could not be directly compared with standard or alternative wound-care modalities, and the present findings should be interpreted primarily as quantitative observational evidence of morphometric healing dynamics during MDT rather than as definitive evidence of comparative therapeutic superiority. Because all cases were derived from a single tertiary care center and MDT was provided as a self-pay service not routinely reimbursed within the national healthcare system, referral and socioeconomic selection bias cannot be excluded, which may limit the generalizability of the findings. Variation in the number of patients attending each session and declining patient numbers in later sessions reduced statistical power for some analyses. Furthermore, the specific reasons for loss to follow-up could not be taken into account. Heterogeneity of comorbid conditions and individual responses to standard wound-care protocols may also have influenced healing trajectories. Morphometric assessments were based on standardized archival photographs, and MDT efficacy was evaluated only by changes in wound surface area; histologic and microvascular parameters were not assessed. Future prospective, controlled, multicenter studies are needed to compare MDT with alternative wound-management strategies, standardize documentation of adjunctive therapies and follow-up outcomes, and confirm these session-based morphometric findings in broader chronic wound populations.

## 5. Conclusions

The present study provides session-based quantitative evidence that MDT is associated with measurable wound-area reduction in chronic wounds. By objectively evaluating wound-area changes across consecutive treatment sessions, this study contributes to a better understanding of the temporal pattern of wound remodeling during MDT. The findings suggest that the most clinically appreciable improvement occurs during the early treatment period, particularly within the first few sessions. Therefore, continuing MDT for at least four sessions may be reasonable when clinically appropriate. Although healing may continue with additional sessions, the rate of improvement appears to decrease over time.

## Figures and Tables

**Table 1:** Wound surface-area reduction rates and stage regression by treatment session.

Session	*n*	Median Reduction % (IQR, Q1–Q3)	≥1 Stage Regression *n* (%)
1	95	3.2 (−2.5 to 12)	17 (18)
2	63	7.0 (2.5 to 14)	6 (9.5)
3	47	8.6 (2.6 to 20)	7 (15)
4	34	8.1 (2.3 to 28)	4 (12)
5	25	7.8 (4.5 to 16)	0 (0)
6	12	4.9 (2.5 to 18)	0 (0)
7	8	15 (2.4 to 24)	1 (13)
8	6	10 (6.9 to 17)	1 (17)
9	4	37 (17 to 58)	0 (0)

Kruskal–Wallis test.

**Table 2:** Pairwise comparisons of wound-area reduction rates during the first five sessions.

Comparison	*p*-Value *
Session 1 vs. Session 2	0.094
Session 1 vs. Session 3	0.029
Session 1 vs. Session 4	0.11
Session 1 vs. Session 5	0.14
Session 2 vs. Session 3	1.00
Session 2 vs. Session 4	1.00
Session 2 vs. Session 5	1.00
Session 3 vs. Session 4	1.00
Session 3 vs. Session 5	1.00
Session 4 vs. Session 5	1.00

Mann–Whitney U Test; * Bonferroni corrected *p*-values.

**Table 3:** Pairwise comparisons of stage regressions.

Comparison	*p*-Value *
4 → 3 vs. 3 → 2	0.74
4 → 3 vs. 2 → 1	0.46
3 → 2 vs. 2 → 1	0.80

Mann–Whitney U Test, * Bonferroni corrected.

**Table 4:** Number at risk, events, censoring, and Kaplan–Meier-estimated cumulative probability for wound area reduction across MDT sessions.

	Number at Risk		Events		Censored		Cumulative Probability	
	≥30% WAR	≥50% WAR	≥30% WAR	≥50% WAR	≥30% WAR	≥50% WAR	≥30% WAR	≥50% WAR
Session 0	95	95	0	0	0	0	0.00	0.00
Session 1	95	95	5	0	29	32	0.05	0.00
Session 2	61	63	9	4	14	14	0.19	0.06
Session 3	38	45	7	3	9	12	0.34	0.13
Session 4	22	30	8	6	6	8	0.58	0.30
Session 5	8	16	2	2	1	5	0.69	0.39
Session 6	5	9	1	0	2	4	0.75	0.39
Session 7	2	5	1	0	0	1	0.87	0.39
Session 8	1	4	1	1	0	3	1.00	0.54

Event: first treatment session at which a lesion achieved ≥30% or ≥50% wound area reduction relative to baseline wound area. Censored: threshold not reached by Session 8 or lost to follow-up. Cumulative probabilities were estimated using the Kaplan–Meier method and are reported as 1 − S(t). *n* = 95 lesions. WAR: wound area reduction; MDT: maggot debridement therapy.

**Table 5:** Logistic regression analysis of the effect of sex and age on 30% and 50% wound-area reduction.

	30% Wound–Area Reduction			50% Wound–Area Reduction		
Variable	OR	CI Lower–Upper	*p*-Value	OR	CI Lower–Upper	*p*-Value
Intercept	0.56	0.027–11	0.70	0.23	0.006–9.7	0.44
Sex	1.8	0.65–4.7	0.27	0.63	0.21–1.9	0.42
Age	0.99	0.95–1.04	0.78	1.00	0.95–1.06	0.89

OR, odds ratio; CI, confidence interval.

## Data Availability

The dataset generated and analyzed during the current study is publicly available in the Zenodo repository: https://doi.org/10.5281/zenodo.19643072 (accessed on 7 June 2026).

## Abbreviations

The following abbreviations are used in this manuscript:

DSLR	Digital Single-Lens Reflex
FDA	Food and Drug Administration
ICC	Intraclass Correlation Coefficient
IQR	Interquartile Range
MDT	Maggot Debridement Therapy
